# Correlation between serum cathepsin S and insulin resistance in type 2 diabetes

**DOI:** 10.3892/etm.2013.1290

**Published:** 2013-09-09

**Authors:** RUO-PING CHEN, AN REN, SHAN-DONG YE

**Affiliations:** Department of Endocrinology, Anhui Provincial Hospital Affiliated to Anhui Medical University, Hefei, Anhui 230001, P.R. China

**Keywords:** cathepsin S, type 2 diabetes, insulin resistance

## Abstract

Cathepsin S (CatS), a proteolytic enzyme, which belongs to the cysteine proteinase family, is associated with atherosclerosis, coronary heart disease, cancer and other diseases. The present study aimed to explore the correlation between serum CatS and insulin resistance (IR) in patients with type 2 diabetes. A total of 51 patients with type 2 diabetes (Group DM) were recruited for this study and 49 healthy individuals were selected as normal controls (Group NC). Blood pressure and body mass index (BMI) were recorded, and serum creatinine, CatS, glycosylated hemoglobin (HbA1c), lipid and insulin levels, and fasting plasma glucose (FPG) levels were measured in all the participants. The homeostatic model assessment index of IR (HOMA-IR) was calculated according to FPG and serum insulin levels. Serum CatS, very low density lipoprotein (VLDL) and triglyceride (TG) levels in Group DM were significantly higher compared with those in Group NC (P=0.000, 0.014 and 0.020, respectively). Significantly positive correlations were identified between CatS levels and VLDL and TG levels, respectively (P<0.05 for both); however, no significant correlations were determined between CatS levels and age, course of disease, blood pressure, cholesterol, BMI, FPG, HbAc1 and HOMA-IR (P>0.05). Further stratification analysis showed that CatS had no association with IR at different HOMA-IR and HbA1c levels. The present study demonstrated that serum CatS, which was significantly increased in patients with type 2 diabetes, had no correlation with IR. This indicates that CatS and IR are independent of each other; however, the precise mechanisms require further investigation.

## Introduction

Insulin resistance (IR) is a reduction in reaction or sensitivity to insulin and was first presented by Reaven in 1988 (1). IR is considered to be the common cause of impaired glucose tolerance, diabetes, obesity, dyslipidemia and hypertensive diseases. IR syndrome is associated with multiple metabolic disorders and was renamed metabolic syndrome (MS) by Zimmet *et al* in 1997 (2). A previous study (3) identified that MS is closely associated with atherosclerosis (AS) and numerous factors, including cytokines, are involved in AS formation. Cathepsin S (CatS) is a cysteine protease, an important lysosomal protease and the predominant cathepsin. CatS is released from primary cultured microglia and regulated by the P2X7 receptor (4), which is associated with matrix metalloproteinases and serine protease, and involved in extracellular proteolytic degradation. In addition, CatS has important physiological functions in the extracellular environment, including degradation of the extracellular matrix (ECM), regulation of growth factors, vascular proliferation of various cytokines, and cell migration, proliferation and apoptosis. Sukhova *et al* (5) demonstrated that CatS expression is increased in atherosclerotic sites. Furthermore, studies have shown that CatS is important for the formation and development of AS (6–9). Type 2 diabetes is a polygenic, hereditary, metabolic disease and the main pathophysiological mechanisms are IR and insufficient insulin secretion.

Since CatS, MS and AS are closely associated with each other, IR is one of the most important pathophysiological mechanisms of type 2 diabetes and AS is one of the pathological features of diabetic vascular complications. However, whether CatS is involved in the pathophysiological mechanisms of type 2 diabetes is yet to be elucidated. A previous study demonstrated that the increased expression of CatS was in parallel with an increase in proinflammatory cytokine levels during the development of autoimmune inflammatory diseases in the non-obese diabetic (NOD) mouse disease model (10). The present study aimed to compare the levels of CatS between patients with type 2 diabetes and healthy individuals, in order to investigate the changes in CatS expression and the possible correlation between CatS and IR.

## Patients and methods

### Patients

A total of 51 patients with type 2 diabetes (Group DM) were recruited from the Department of Endocrinology, Anhui Provincial Hospital Affiliated to Anhui Medical University (Hefei, China). All patients (age range, 38–80 years) coincided with the 1999 World Health Organization standards for diabetes diagnosis and classification (11). Exclusion criteria comprised patients who had heart, liver or pancreatic diseases; primary or secondary renal diseases (with the exception of diabetic nephropathy); inflammatory tumor or immunity diseases; impaired glucose tolerance induced by glucocorticoid and steroidal agents; and acute complications or serious chronic complications of diabetes mellitus, including ketoacidosis, hyperosmolar coma and lactic acidosis. Forty-nine healthy individuals without diabetes, infection, tumors and autoimmune diseases were chosen as the normal controls (Group NC; 25 males and 24 females). A total of 51 diabetic patients in DM group included 26 males and 25 females. The study was approved by the local research ethics committee (Anhui Provincial Hospital, Anhui, China) and written informed consent was obtained from all patients before the study commenced.

### Methods

The gender, age, height, body weight, systolic pressure and diastolic pressure of the patients were recorded, and the body mass index (BMI) and the homeostatic model assessment index of IR (HOMA-IR) were calculated in all participants. Venous blood samples were collected to measure serum creatinine, cholesterol (TCH), triglyceride (TG), very low density lipoprotein (VLDL), fasting plasma glucose (FPG), insulin and glycosylated hemoglobin A1c (HbA1c) levels following >8 h of overnight fasting. Simultaneously, a serum sample was collected and preserved at −80°C to test for the expression of CatS.

Serum CatS levels were determined using a sandwich enzyme immunoassay (ELISA; Krka, d.d., Novo mesto, Riga, Slovenia; Riga, Slovenia; Human Cathepsin S) according to the manufacturer’s instructions. Serum samples were diluted 1:1 with 0.9% physiological saline, and then added to the pre-coated ELISA plate (100 *μ*l per well). Plates were incubated overnight at 4°C, washed and incubated with conjugated solution for 2 h at 37°C. Plates were then developed by treatment with 3,3′,5,5′-tetramethylbenzidine (TMB) and H_2_SO_4_ solution according to the manufacturer’s instructions of the ELISA kit. The serum concentrations of CatS were determined from standard curves derived from the provided calibrators, and ranged from 0.1 to 4.4 nmol/l. The serum biochemical indices were measured using an automatic biochemical analyzer (Hitachi no. 7600-020; Hitachi High-Technologies Corporation, Tokyo, Japan).

### Statistical analysis

Clinical data and serum Cat S levels were analyzed using the nonparametric Wilcoxon test, linear and multiple linear regression. The HOMA-IR was converted into logarithms to achieve approximate normality. P<0.05 was considered to indicate a statistically significant difference.

## Results

### Comparison of the clinical data between Group NC and Group DM

As shown in Table I, a comparison of the clinical data between Group NC and Group DM indicated no significant differences in age, and serum creatinine and TCH levels. However, the CatS, FPG, VLDL and TG levels in Group DM were significantly higher than those in Group NC (P=0.000, 0.000, 0.014 and 0.020, respectively).

### Correlation between serum CatS levels and various clinical indices

Changes in the serum levels of CatS were significantly associated with TG and VLDL levels (P<0.05 for both); however, no significant correlations were identified between serum CatS levels and age, disease course, blood pressure, TCH, BMI, FPG, Cr, HbAc1 and HOMA-IR (Table II).

### Correlation between serum CatS levels and clinical data in Group DM at different HOMA-IR levels

The third quartile is 2.095. Regardless of whether the HOMA-IR level was low (<2.095) or high (≥2.095), there no significant correlations were identified between the serum levels of CatS and age, disease course, blood pressure, TCH, BMI, FPG, Cr, TG, VLDL, HbAc1 and HOMA-IR (Tables III and IV, respectively).

### Correlation between serum CatS level and clinical data in Group DM at different HbA1c levels

When the HbA1c level was <9% (Table V), there were no significant correlations between serum CatS and age, disease course, blood pressure, TCH, BMI, FPG, Cr, TG, VLDL, HbAc1 and HOMA-IR. However, when the HbA1c levels were ≥9%, significant correlations were identified between serum CatS levels and disease course, TG and VLDL levels (P=0.025, 0.021 and 0.022, respectively; Table VI).

### Correlation between serum CatS levels and clinical data in Group DM at different BMI levels

At a BMI of <25 kg/m^2^, the serum CatS level was significantly associated with disease course, and TG and VLDL levels (P=0.033, 0.027 and 0.023, respectively; Table VII). However, no significant correlations were determined between those factors when the BMI was ≥25 kg/m^2^ (Table VII). In addition, there were no significant correlations between serum CatS and age, blood pressure, TCH, BMI, FPG, Cr, HbAc1 and HOMA-IR regardless of whether the BMI was <25 or ≥25 kg/m^2^.

## Discussion

CatS was first identified in hematopoietic cell endosomes, which belong to the non-glycosylated papain-like single-chain protease family, and demonstrates proteolytic and endopeptidase effects. Numerous studies have shown that CatS is associated with AS, coronary heart disease and cancer. However, few studies have identified a correlation between CatS and diabetes. In animal models, deletion of the CatS gene resulted in immunity to type 1 diabetes mellitus in certain NOD mice (12). The present study indicated that CatS levels were significantly higher in patients with type 2 diabetes than those in the normal controls, and this is consistent with the findings of Liu *et al* (13). The current study suggests that CatS may be involved in the occurrence and development of diabetes.

IR is a one of the key pathophysiological mechanisms of type 2 diabetes and serum CatS levels are elevated in patients with type 2 diabetes. Therefore, it is necessary to investigate the association between serum CatS concentration and IR in patients with type 2 diabetes. The hyperinsulinemic-euglycemic clamp test is the gold standard for evaluating IR. In addition, BMI, waist circumference, waist to hip ratio, fasting insulin, fasting blood glucose, Shima Soku used the insulin action index (IAI) and HOMA-IR, may also be used as auxiliary indices for evaluating IR. The present study selected BMI and HOMA-IR as the indices of IR. Through hierarchical analysis, no significant correlations between serum CatS and BMI and HOMA-IR were detected. This suggests that HOMAIR, serum CatS and IR are independent of each other, and that CatS may be involved in the occurrence and development of diabetes as an independent factor.

Due to reductions in lipoprotein lipase activity, the TG levels readily increase and VLDL clearance decreases after eating in patients with diabetes, and the accumulation of VLDL increases. Increases in TG levels in the skeletal muscle are also considered to be one of the causes of IR (14). The present study identified that serum CatS levels were significantly associated with TG and VLDL levels when the HbA1c level was >9% or the BMI was <25 kg/m^2^, respectively. The exact mechanisms responsible for this remain unclear, but may be related to postprandial glucose. In addition, it has been indicated that CatS promotes fat formation, particularly in the early stages of adipocyte differentiation, which suggests that CatS may be involved in the pathophysiological mechanisms of lipid metabolism.

The duration of diabetes (disease course) is correlated with decreased B cell function. The present study demonstrates a significant correlation between serum CatS and diabetes course when the HbA1c level is >9% or the BMI is <25 kg/m^2^. However, the precise mechanisms require further investigation.

It has been demonstrated that patients with type 2 diabetes have a low-grade inflammatory state *in vivo* (15). Inflammation may cause IR and is involved in associated diseases, including diabetes, metabolic syndrome and AS. Jobs *et al* (16) identified that serum CatS levels were associated with C-reactive protein, which is an important indicator of inflammation. In addition, Naour *et al* (17) indicated that CatS may also be involved in the energy balance in adipose tissue and circulation. These studies suggest that serum CatS may be independent of IR, and involved in the occurrence and development of diabetes through certain pathways, including the inflammatory response.

A limitation of the present study is that the sample size was relatively low, which may have affected the HOMA-IR results. In addition, more precise methods for the assessment of IR, including glucose clamp experiments, could have been used. In addition, the related inflammatory indices and postprandial blood glucose levels require analysis

In conclusion, the present study demonstrates that serum CatS levels are significantly increased in patients with type 2 diabetes, which reflects their inflammatory state and shows no significant correlation with IR. The precise mechanisms responsible for this require further investigation.

## Figures and Tables

**Table I.** Comparison of the clinical data between Group NC and Group DM.

Clinical index	Group DM	Group NC	P-value
Age (year)	55.84±10.91	60.43±1.87	0.065
Serum Cr (*μ*mol/l)	79.76±43.35	79.85±14.51	0.045
TCH (mmol/l)	4.96±1.24	4.54±0.91	0.109
VLDL (mmol/l)	0.98±0.63	0.78±0.74	0.014
TG (mmol/l)	2.18±1.43	1.72±1.66	0.020
FPG (mmol/l)	11.53±3.88	5.19±0.74	0.000
CatS (nmol/l)	0.46±0.01	0.39±0.01	0.000

Group NC, normal control; Group DM, patients with type 2 diabetes; Cr, creatinine; TCH, cholesterol; VLDL, very low density lipoprotein; TG, triglyceride; FPG, fasting plasma glucose; CatS, cathepsin S.

**Table II.** Correlation between serum CatS and other clinical indices in Group DM.

Clinical index	Mean ± sd	CatS		
		R^2^	Coefficient	P-value
Age (year)	55.84±10.91	0.024	0.001	0.274
Course (year)	5.59±0.74	0.074	−0.005	0.054
SBP (mmHg)	134.76±3.21	0.008	0.000	0.524
DBP (mmHg)	77.72±1.16	0.005	−0.001	0.623
BMI (kg/m^2^)	23.28±0.44	0.033	0.005	0.201
FPG (mmol/l)	11.53±3.88	0.018	−0.003	0.348
Serum Cr (*μ*mol/l)	79.76±43.35	0.036	0.000	0.180
TCH (mmol/l)	4.96±1.24	0.015	0.009	0.398
TG (mmol/l)	2.18±1.43	0.108	0.021	0.019
VLDL (mmol/l)	0.98±0.63	0.113	0.048	0.016
HbA1c (%)	12.59±0.67	0.000	0.000	0.895
HOMA-IR	1.62±0.11	0.002	−0.006	0.732

CatS, cathepsin S; Group DM, patients with type 2 diabetes; SBP, systolic blood pressure; DBP, diastolic blood pressure; BMI, body mass index; FPG, fasting plasma glucose; Cr, creatinine; TCH, cholesterol; TG, triglyceride; VLDL, very low density lipoprotein; HbAlc, glycosylated hemoglobin; HOMA-IR, homeostatic model assesment index of insulin resistance.

**Table III.** Correlation between serum CatS levels and clinical data in Group DM at a HOMA-IR level <2.095.

Clinical index	Mean ± SD	CatS		
		R^2^	Coefficient	P-value
Age (year)	55.03±10.29	0.009	0.001	0.577
Course (year)	5.95±5.42	0.070	−0.005	0.110
SBP (mmHg)	135.74±24.42	0.004	0.000	0.724
DBP (mmHg)	76.63±8.52	0.009	−0.001	0.577
BMI (kg/m^2^)	22.67±2.93	0.037	0.006	0.249
FPG (mmol/l)	10.95±3.79	0.016	−0.003	0.443
Serum Cr (*μ*mol/l)	78.33±45.38	0.018	0.000	0.423
TCH (mmol/l)	4.86±1.27	0.016	0.009	0.444
TG (mmol/l)	2.04±1.38	0.098	0.021	0.055
VLDL (mmol/l)	0.93±0.62	0.094	0.047	0.061
HbA1c (%)	11.55±3.24	0.037	−0.006	0.245
HOMA-IR	1.28±0.49	0.015	0.023	0.470

CatS, cathepsin S; Group DM, patients with type 2 diabetes; SBP, systolic blood pressure; DBP, diastolic blood pressure; BMI, body mass index; FPG, fasting plasma glucose; Cr, creatinine; TCH, cholesterol; TG, triglyceride; VLDL, very low density lipoprotein; HbAlc, glycosylated hemoglobin; HOMA-IR, homeostatic model assesment index of insulin resistance.

**Table IV.** Correlation between serum CatS levels and clinical data in Group DM at a HOMA-IR level ≥2.095.

Clinical index	Mean ± SD	CatS		
		R^2^	Coefficient	P-value
Age (year)	58.23±12.68	0.132	0.002	0.223
Course (year)	4.54±4.94	0.101	−0.006	0.290
SBP (mmHg)	131.92±18.43	0.053	0.001	0.432
DBP (mmHg)	80.92±6.77	0.003	0.001	0.857
BMI (kg/m^2^)	25.08±3.07	0.072	0.008	0.375
FPG (mmol/l)	13.20±3.76	0.023	−0.003	0.622
Serum Cr (*μ*mol/l)	83.95±38.15	0.241	0.001	0.089
TCH (mmol/l)	5.26±1.15	0.018	0.010	0.664
TG (mmol/l)	2.59±1.58	0.191	0.024	0.135
VLDL (mmol/l)	1.15±0.67	0.231	0.061	0.097
HbA1c (%)	15.65±7.02	0.165	0.005	0.169
HOMA-IR	2.63±0.58	0.182	−0.063	0.146

CatS, cathepsin S; Group DM, patients with type 2 diabetes; SBP, systolic blood pressure; DBP, diastolic blood pressure; BMI, body mass index; FPG, fasting plasma glucose; Cr, creatinine; TCH, cholesterol; TG, triglyceride; VLDL, very low density lipoprotein; HbAlc, glycosylated hemoglobin; HOMA-IR, homeostatic model assesment index of insulin resistance.

**Table V.** Correlation between serum CatS levels and clinical data in Group DM with a HbA1c level <9%.

Clinical index	Mean ± SD	CatS		
		R^2^	Coefficient	P-value
Age (year)	58.60±12.73	0.019	0.001	0.627
Course (year)	6.40±5.15	0.008	−0.002	0.753
SBP (mmHg)	136.47±23.83	0.207	0.002	0.088
DBP (mmHg)	79.40±8.75	0.008	0.001	0.745
BMI (kg/m^2^)	23.24±2.54	0.032	0.007	0.523
FPG (mmol/l)	8.60±2.59	0.103	0.012	0.244
Serum Cr (*μ*mol/l)	88.28±52.59	0.002	−8.3E-005	0.874
TCH (mmol/l)	5.19±1.46	0.003	0.004	0.840
TG (mmol/l)	2.21±1.48	0.049	0.015	0.427
VLDL (mmol/l)	0.98±0.63	0.066	0.040	0.355
HbA1c (%)	7.79±0.99	0.082	0.028	0.302
HOMA-IR	1.50±0.69	0.004	−0.009	0.820

CatS, cathepsin S; Group DM, patients with type 2 diabetes; SBP, systolic blood pressure; DBP, diastolic blood pressure; BMI, body mass index; FPG, fasting plasma glucose; Cr, creatinine; TCH, cholesterol; TG, triglyceride; VLDL, very low density lipoprotein; HbAlc, glycosylated hemoglobin; HOMA-IR, homeostatic model assesment index of insulin resistance.

**Table VI.** Relationship between serum CatS levels and clinical data in Group DM with a HbA1c level ≥9%.

Clinical index	Mean ± SD	CatS		
		R^2^	Coefficient	P-value
Age (year)	54.69±10.03	0.018	0.001	0.429
Course (year)	5.25±5.38	0.139	−0.006	0.025
SBP (mmHg)	134.06±22.85	0.007	0.000	0.638
DBP (mmHg)	77.03±8.06	0.034	−0.002	0.281
BMI (kg/m^2^)	23.30±3.37	0.036	0.005	0.265
FPG (mmol/l)	12.75±3.69	0.042	−0.005	0.231
Serum Cr (*μ*mol/l)	76.21±39.18	0.106	0.001	0.053
TCH (mmol/l)	4.87±1.15	0.018	0.010	0.435
TG (mmol/l)	2.16±1.44	0.146	0.023	0.021
VLDL (mmol/l)	0.99±0.64	0.144	0.052	0.022
HbA1c (%)	14.60±4.27	0.026	0.003	0.346
HOMA-IR	1.68±0.82	0.000	−0.002	0.900

CatS, cathepsin S; Group DM, patients with type 2 diabetes; SBP, systolic blood pressure; DBP, diastolic blood pressure; BMI, body mass index; FPG, fasting plasma glucose; Cr, creatinine; TCH, cholesterol; TG, triglyceride; VLDL, very low density lipoprotein; HbAlc, glycosylated hemoglobin; HOMA-IR, homeostatic model assesment index of insulin resistance.

**Table VII.** Correlation between serum CatS levels and clinical data in Group DM with a BMI <25 kg/m^2^.

Index	Mean ± SD	CatS		
		R^2^	Coefficient	P-value
Age (year)	56.06±10.04	0.017	0.001	0.448
Course (year)	6.40±5.83	0.126	−0.005	0.033
SBP (mmHg)	129.75±23.22	0.008	0.000	0.613
DBP (mmHg)	76.11±8.31	0.044	−0.002	0.219
BMI (kg/m^2^)	21.68±1.99	0.104	0.013	0.055
FPG (mmol/l)	11.78±4.04	0.062	−0.005	0.143
Serum Cr (*μ*mol/l)	79.19±47.31	0.016	0.000	0.466
TCH (mmol/l)	4.87±1.38	0.064	0.014	0.137
TG (mmol/l)	2.06±1.62	0.136	0.018	0.027
VLDL (mmol/l)	0.93±0.71	0.142	0.042	0.023
HbA1c (%)	11.94±3.99	0.040	−0.004	0.243
HOMA-IR	1.54±0.79	0.000	0.000	0.981

CatS, cathepsin S; Group DM, patients with type 2 diabetes; SBP, systolic blood pressure; DBP, diastolic blood pressure; BMI, body mass index; FPG, fasting plasma glucose; Cr, creatinine; TCH, cholesterol; TG, triglyceride; VLDL, very low density lipoprotein; HbAlc, glycosylated hemoglobin; HOMA-IR, homeostatic model assesment index of insulin resistance.

**Table VIII.** Correlation between serum CatS levels and clinical data in Group DM with a BMI ≥25 kg/m^2^.

Clinical index	Mean ± SD	CatS		
		R^2^	Coefficient	P-value
Age (year)	55.33±13.13	0.038	0.002	0.485
Course (year)	3.63±3.01	0.008	−0.003	0.754
SBP (mmHg)	146.80±17.61	0.007	0.001	0.767
DBP (mmHg)	81.60±6.92	0.016	0.002	0.654
BMI (kg/m^2^)	27.12±1.56	0.007	0.006	0.766
FPG (mmol/l)	10.92±3.53	0.004	0.002	0.816
Serum Cr (*μ*mol/l)	81.12±33.34	0.177	0.001	0.119
TCH (mmol/l)	5.18±0.82	0.051	−0.033	0.418
TG (mmol/l)	2.47±0.83	0.146	0.023	0.021
VLDL (mmol/l)	1.11±0.37	0.118	0.110	0.210
HbA1c (%)	14.16±6.16	0.053	0.004	0.409
HOMA-IR	1.83±0.74	0.024	−0.025	0.580

CatS, cathepsin S; Group DM, patients with type 2 diabetes; SBP, systolic blood pressure; DBP, diastolic blood pressure; BMI, body mass index; FPG, fasting plasma glucose; Cr, creatinine; TCH, cholesterol; TG, triglyceride; VLDL, very low density lipoprotein; HbAlc, glycosylated hemoglobin; HOMA-IR, homeostatic model assesment index of insulin resistance.
